# Demoralization profiles and their association with active aging in Chinese older adults with disabilities: a latent profile analysis

**DOI:** 10.3389/fpubh.2025.1715566

**Published:** 2025-12-09

**Authors:** Lulu Wu, Ziqing Qi, Yue Zhang, Dan Zhang, Jia Wang, Yali Mao, Ruting Wang, Annuo Liu

**Affiliations:** School of Nursing, Anhui Medical University, Hefei, China

**Keywords:** demoralization syndrome, older adults with disabilities, active aging, latent profile analysis, psychological adaptation

## Abstract

**Objective:**

To identify latent profiles of demoralization among older adults with disabilities, analyze their influencing factors, and examine their associations with active aging.

**Methods:**

From February to July 2025, a convenience sample of 411 older adults with disabilities was recruited from a tertiary hospital in Anhui Province, China. Data were collected using a general information questionnaire, the Chinese version of the Demoralization Scale, and the Active Aging Scale. Latent profile analysis (LPA) was performed based on demoralization subscale scores. Univariate and multinominal analyses were employed to investigate the influencing factors, and the Kruskal–Wallis *H* test was used to compare differences in active aging levels across the profiles.

**Results:**

The prevalence of demoralization syndrome was 49.1%. LPA identified three distinct profiles: the Well-Adapted Group (53.3%), the Disheartened-Helpless Group (23.8%), and the Fully Demoralized Group (22.9%). The Kruskal–Wallis *H* test revealed significant differences among the profiles (*p* < 0.001), indicating a negative correlation between active aging levels and demoralization severity. The Fully Demoralized Group scored significantly lower on active aging than the other two groups.

**Conclusion:**

Nearly half of the older adults with disabilities experienced demoralization, with heterogeneous subgroups identified. The active aging status of demoralized subgroups requires urgent attention. These findings suggest the need for targeted interventions tailored to the characteristics of each profile to improve mental health and promote active aging in this population.

## 1 Introduction

Against the backdrop of accelerating global population aging, active aging has progressively emerged as a key strategy to address associated societal challenges. This concept, introduced by the World Health Organization in 2002, emphasizes optimizing the three pillars of health, participation, and security to enhance the quality of life for older persons comprehensively (1). This inclusive concept of active aging is not limited to healthy older adults but explicitly encompasses vulnerable groups, including those with disabilities. By the end of 2022, the population aged 60 and above (the statutory standard for defining older adults in China) had reached 280 million (2), among whom approximately 44 million were disabled or partially disabled, accounting for 15.7% of the total older population (3). Disabled older adults, as a vulnerable segment, face many challenges in pursuing active aging. Physical impairments restrict their capacity for social participation, threaten psychological well-being, and result in significantly lower levels of active aging compared to their healthy counterparts (4, 5). Therefore, focusing on the health status of this substantial population is crucial for achieving the overarching goal of active aging (6).

In addition to physical dysfunction, older adults with disabilities commonly face various psychological issues (7, 8), which can consequently affect the maintenance or improvement of their physical function (9). Demoralization syndrome is one such issue. It is a distinct psychological distress state characterized by low morale, hopelessness, subjective incompetence, and a loss of meaning and purpose (10). Studies from 2014 to 2020 reported an average prevalence rate of 24 to 35% (11). Previous research has linked demoralization with various factors, such as chronic disease, severity of physical symptoms, functional impairment, mobility limitations, body image changes, and social isolation (11–14). Disabled older adults, who often experience a confluence of these risk factors, are undoubtedly a high-risk group for developing demoralization syndrome. More critically, the sense of meaninglessness and subjective incompetence in demoralization can undermine the willingness and motivation for social participation and health self-management (15). This may hinder the realization of active aging.

According to the theory of stress and coping, the cognitive appraisal an individual makes when facing stress is a core factor influencing their coping strategies and adaptive outcomes (16). For older adults with disabilities, an objective appraisal of their own condition is crucial for integrating resources and adapting to change. While some individuals successfully adapt and reconstruct meaning in life, a significant proportion are unable to adjust to functional loss and social role exit. They develop feelings of disheartenment and hopelessness, then sink into demoralization (17). Research indicates that demoralization not only reduces quality of life and exacerbates physical symptoms but also leads to decreased treatment adherence and an increased risk of suicide (18).

Despite the significant impact of demoralization among older adults with disabilities, research on this condition within the population remains in its early stages, and its prevalence patterns are not yet fully understood. Existing studies are hampered by inconsistent assessment tools, leading to poor comparability of results. Furthermore, the reliance on total scores to assess demoralization severity fails to reveal the heterogeneity within the group, consequently hindering the development of targeted interventions (19–21). In fact, the clinical presentation of demoralization syndrome is highly heterogeneous, with significant potential variations in symptom combinations, severity, and underlying psychological experiences among different individuals (22). Latent Profile Analysis (LPA) is a person-centered approach that identifies distinct subgroups by analyzing individuals’ response patterns to observed variables. It provides a deeper analysis of individual differences and is well-suited for exploring the heterogeneity in demoralization manifestations (23, 24).

To address these gaps, this study aims to use LPA to identify potential demoralization profiles among Chinese older adults with disabilities, analyze differences in demographic and disease characteristics across these profiles, and further examine the relationship between profile membership and active aging. The findings are expected to provide an empirical basis for developing targeted psychological interventions, ultimately aiming to enhance mental health and active aging levels in this population.

## 2 Materials and methods

### 2.1 Study design and participants

This cross-sectional study utilized a convenience sampling method to conduct questionnaire surveys among older adults with disabilities at a tertiary hospital in Hefei City, Anhui Province, from February to July 2025. Inclusion criteria were: (1) age ≥ 60 years; (2) Barthel Index score ≤ 95, with the disability lasting for at least 6 months; (3) provision of informed consent and voluntary participation in the survey. Exclusion criteria were: (1) presence of severe physical illnesses such as advanced malignant tumors or end-stage renal disease, or being in the acute phase of an illness, rendering participants unable to cooperate; (2) a clinical diagnosis of mental illness or presence of cognitive impairment; (3) severe hearing, visual, or speech impairments preventing effective communication. Three graduate students received unified training and administered face-to-face questionnaires to the participants, ensuring each interview lasted approximately 15 to 20 min.

### 2.2 Sample size

The sample size was determined based on the statistical methods employed in the study, namely Latent Profile Analysis (LPA) and multinominal logistic regression. LPA requires a minimum of 50 samples per potential class (25). Anticipating 2 to 5 latent classes, the estimated sample size requirement ranged from 100 to 250. A review of previous studies utilizing LPA revealed that sample sizes varied widely, from 200 to over 1,000 (26–28). For the multinominal logistic regression component, Kendall’s method was used for sample size estimation. As a rule of thumb for cross-sectional studies, the sample size should be at least 5 to 10 times the number of variables included (29). This study incorporated 29 variables. Accounting for a 15% rate of invalid questionnaires, the calculated sample size range was 171 to 342. Therefore, by considering participant availability and drawing on the precedent of previous research, a final sample size of 411 older adults with disabilities was included in the study.

### 2.3 Measurement

#### 2.3.1 Sociodemographic and health-related characteristics

The questionnaire was designed by the researchers based on a review of relevant domestic and international literature. Sociodemographic characteristics included age, gender, religious affiliation, place of residence, living arrangement, educational level, pension status, monthly household income per capita, number of living children, primary caregiver, marital status, and healthcare payment method. Health-related attributes included the number of chronic diseases, the Barthel Index score, disability duration, cause of disability, and self-rated health status.

#### 2.3.2 Barthel index scale

The Barthel Index, designed by Dorothea Barthel and Florence Mahoney (30) in 1965, was used for assessment. It is the most widely used and extensively researched scale in clinical practice for evaluating activities of daily living (ADL), with well-established reliability and validity (5). The scale assesses 10 items, including feeding, bathing, grooming, dressing, and bowel/bladder control, etc. Each item is rated across four levels: complete independence, requires partial assistance, requires substantial assistance, and complete dependence. The total score ranges from 0 to 100. A score of 100 indicates normal ADL ability, complete self-care, and no need for assistance. A score between 61 and 99 indicates mild disability, requiring partial assistance. A score between 41 and 60 indicates moderate disability, requiring substantial assistance. A score below 40 indicates severe disability, implying an inability to perform ADL independently or a need for full-time care. The total score reflects an individual’s level of self-care ability. In this study, the scale was used to measure the general functional status of older adults with disabilities. Individuals requiring assistance or being unable to complete at least one ADL item were defined as having a disability. The Cronbach’s *α* coefficient for the scale in this study was 0.810.

#### 2.3.3 Chinese version of the demoralization syndrome scale

The Demoralization Scale was originally developed by Kissane et al. (31), and subsequently adapted into Chinese by Hong et al. (32), with further cultural adaptation performed by Liu et al. (33), to assess demoralization in patients. The scale consists of 24 items encompassing five dimensions: loss of meaning, dysphoria, disheartenment, helplessness, and sense of failure. Each item is rated on a 5-point Likert scale ranging from 0 (strongly disagree) to 4 (strongly agree). The total score ranges from 0 to 96, with higher scores indicating more severe demoralization. A total score greater than 30 indicates the presence of demoralization syndrome. In this study, the Cronbach’s *α* coefficient for the scale was found to be 0.958.

#### 2.3.4 Active aging scale

The Active Aging Scale (AAS) was used to measure the level of active aging in older adults. The AAS was originally developed by Thai scholar Thanakwang et al. (34) based on the active aging theory. The Chinese version was subsequently created by Zhang et al. (35) from the Chinese research team, who performed translation, back-translation, and cultural adaptation following the Brislin translation model. The AAS comprises 36 items across seven dimensions: self-reliance, active learning and social integration, growing spiritual wisdom, establishing financial security, maintaining a healthy lifestyle, contributing positively to society, and being a role model. Items are rated on a 4-point Likert scale. The total score ranges from 36 to 144, with higher scores indicating a higher level of active aging. In this study, the Cronbach’s *α* coefficient for the scale was found to be 0.902.

### 2.4 Data collection

Data collection was carried out by three graduate students (LW, ZQ, and DZ) who received standardized training before the commencement of data collection. The training covered the study’s objectives, familiarity with the scales, and ensuring consistency in the interpreters’ understanding of the items. Discussions were held to standardize the language and phrasing used to ask questions, ensuring they were easily understandable for older adults.

Before data collection, the researchers explained the purpose of the study to eligible older adults with disabilities. Paper-based questionnaires were administered only to participants who provided informed consent. Participants with literacy skills completed the questionnaires independently, while researchers remained present to promptly address any questions regarding the items. For participants without literacy skills, the researchers read the questions aloud and recorded their responses. If a participant expressed unwillingness to continue during the survey process, the interview was stopped immediately, and the corresponding questionnaire was deemed invalid. After completion, researchers checked the questionnaires on-site for any missing items to ensure data completeness.

Out of 433 questionnaires distributed, 411 valid responses were obtained, resulting in an effective response rate of 94.9%.

### 2.5 Statistical analysis

Data entry was performed by researcher LW using Microsoft Excel 2021 and verified by another researcher, YZ. In this study, SPSS 25.0 and Mplus 8.3 were used for the statistical analysis, and the results were statistically significant as bilateral (*p* < 0.05).

SPSS 25.0 was used for statistical description of the study population. The count data were described using frequencies and percentages, while total scores across scales were presented using medians and quartiles. Kruskal–Wallis *H* test and chi-square test were employed for intergroup comparisons. The Barthel index, demoralization, and active aging scores all deviated from a normal distribution (*p* < 0.05).

Latent Profile Analysis (LPA) was performed on the five dimensions of demoralization syndrome using Mplus 8.3. The analysis began by fitting a one-profile model, and the number of profiles was incrementally increased. The following indices were used to evaluate the LPA models and select the optimal solution: Akaike Information Criterion (AIC), Bayesian Information Criterion (BIC), adjusted Bayesian Information Criterion (aBIC), entropy, the Lo–Mendell–Rubin adjusted likelihood ratio test (LMR), and the Bootstrap Likelihood Ratio Test (BLRT) (36). Lower values of AIC, BIC, and aBIC indicate better model fit. Entropy was used to assess classification accuracy, with a value ≥ 0.80 indicating that approximately 90% of cases were correctly classified. The LMR and BLRT were used to compare the fit of models with adjacent numbers of profiles; significant *p*-values (*p* < 0.05) suggest that the model with n profiles provides a statistically superior fit to the model with n-1 profiles (37).

Subsequently, univariate analysis and multinominal logistic regression were performed using SPSS 25.0 to explore the factors influencing latent profile membership. Finally, the Kruskal–Wallis *H* test was used to compare the total active aging scores across the identified latent profiles (38).

### 2.6 Ethics statement

This study was conducted in accordance with the Declaration of Helsinki and received approval from the Ethics Committee of Anhui Medical University (No. 82240101). The investigators provided eligible patients with both verbal and written information regarding the study procedures. Patients were informed that their participation was entirely voluntary, that they could withdraw at any time without affecting their medical care, and that the privacy of their data would be protected. Written informed consent was obtained from all participants before data collection.

## 3 Results

### 3.1 Common method bias tests

In this study, we assessed the risk of common method bias. This bias can arise from using similar measurement methods across variables and may lead to spurious correlations. To mitigate this risk, we employed Harman’s single-factor test by performing an exploratory factor analysis on all items from the scales included in the study. The analysis yielded 16 factors with eigenvalues greater than 1. The primary factor accounted for 28.2% of the variance, which is below the 40% threshold (38), indicating that the data in this study were not substantially threatened by common method bias.

### 3.2 Characteristics of older adults with disabilities

The median age of the older adults with disabilities in this study was 68(65, 75), with an age range of 60 to 87 years. Slightly more than half of the participants were male (52.6%), the majority were married (71.5%), had no religious affiliation (84.7%), and had attained a junior high or high school education level (61.1%). In terms of disability severity, mild and moderate disability accounted for 66.2 and 30.9% of the sample, respectively. The majority (61.8%) had lived with a disability for less than 6 years. Disease and physical aging were reported as the primary causes of disability for 72.5% of participants, and 92.0% reported having one or more chronic conditions. Further details are provided in Table 1.

**Table 1:** Sociodemographic characteristics of research subjects and intergroup comparisons (*n* = 411).

Characteristics [*n*(%)]	Overall (*n* = 411)	Classification of latent profiles			$x^{2}$ /*H*	*P*
		Class 1 [%] *n* = 219	Class 2 [%] *n* = 98	Class 3 [%] *n* = 94		
Gender						
Male	216(52.6)	115(52.5)	58(59.2)	43(45.7)	3.476	0.176
Female	195(47.4)	104(47.5)	40(40.8)	51(54.3)		
Age (years)						
60~	237(57.7)	126(57.5)	60(61.2)	51(54.3)	1.871	0.759
70~	127(30.9)	67(30.6)	30(30.6)	30(31.9)		
80~	47(11.4)	26(11.9)	8(8.2)	13(13.8)		
Religion						
No	348(84.7)	190(86.8)	71(72.4)	87(92.6)	16.514	<0.001^***^
Yes	63(15.3)	29(13.2)	27(27.6)	7(7.4)		
Marital status						
Unmarried	11(2.7)	7(3.2)	2(2.0)	2(2.1)	4.797	0.570
Married	294(71.5)	153(69.9)	76(77.6)	65(69.2)		
Divorced	25(6.1)	16(7.3)	2(2.0)	7(7.4)		
Widowed	81(19.7)	43(19.6)	18(18.4)	20(21.3)		
Education level						
Primary school or below	78(19.0)	28(12.8)	28(28.6)	22(23.5)	32.152	<0.001^***^
Junior school	126(30.7)	65(29.7)	37(37.8)	24(25.5)		
High school or vocational school	125(30.4)	64(29.2)	26(26.5)	35(37.2)		
College/university or higher	82(19.9)	62(28.3)	7(7.1)	13(13.8)		
Residence						
Rural	126(30.7)	52(23.7)	46(46.9)	28(29.8)	20.756	<0.001^***^
Towns	176(42.8)	97(44.3)	32(32.7)	47(50.0)		
Cities	109(26.5)	70(32.0)	20(20.4)	19(20.2)		
Living status						
Living alone	56(13.6)	35(16.0)	13(13.3)	8(8.5)	14.556	0.006^**^
With families	337(82.0)	177(80.8)	84(85.7)	76(80.9)		
Other	18(4.4)	7(3.2)	1(1.0)	10(10.6)		
Pension status						
No	202(49.1)	92(42.0)	57(58.2)	53(56.4)	9.621	0.008^**^
Yes	209(50.9)	127(58.0)	41(41.8)	41(43.6)		
Monthly household income per capita (RMB)						
≤3,000	117(28.5)	39(17.8)	49(50.0)	29(30.9)	39.970	<0.001^***^
3,001–5,000	172(41.8)	96(43.8)	35(35.7)	41(43.6)		
≥5,001	122(29.7)	84(38.4)	14(14.3)	24(25.5)		
Number of living children						
None	15(3.7)	7(3.2)	1(1.0)	7(7.4)	8.925	0.178
1	84(20.4)	46(21.0)	18(18.4)	20(21.3)		
2	173(42.1)	96(43.8)	38(38.8)	39(41.5)		
≥3	139(33.8)	70(32.0)	41(41.8)	28(29.8)		
Primary caregiver						
Self-care	76(18.5)	46(21.0)	17(17.3)	13(13.8)	15.437	0.004^**^
Spouse/ Children	255(62.0)	120(54.8)	73(74.5)	62(66.0)		
Other	80(19.5)	53(24.2)	8(8.2)	19(20.2)		
Healthcare payment method						
Self-pay	56(13.6)	20(9.1)	16(16.3)	20(21.3)	9.884	0.042^*^
Resident insurance	267(65.0)	146(66.7)	64(65.3)	57(60.7)		
Employee insurance	88(21.4)	53(24.2)	18(18.4)	17(18.0)		
Chronic diseases						
None	33(8.0)	18(8.2)	12(12.3)	3(3.2)	5.976	0.201
1–2	233(56.7)	127(58.0)	50(51.0)	56(59.6)		
≥3	145(35.3)	74(33.8)	36(36.7)	35(37.2)		
Disability duration						
6 months~	44(10.7)	24(11.0)	12(12.3)	8(8.5)	20.156	0.010^*^
1 year~	79(19.2)	44(20.0)	12(12.3)	23(24.5)		
3 years~	131(31.9)	76(34.7)	34(34.6)	21(22.4)		
6 years~	94(22.9)	51(23.3)	26(26.5)	17(18.0)		
≥9 years	63(15.3)	24(11.0)	14(14.3)	25(26.6)		
Cause of disability						
Congenital disability	7(1.7)	2(0.9)	1(1.0)	4(4.3)	25.555	<0.001^***^
Illness	178(43.3)	75(34.3)	48(49.0)	55(58.5)		
Accident	106(25.8)	64(29.2)	22(22.4)	20(21.3)		
Aging	120(29.2)	78(35.6)	27(27.6)	15(15.9)		
Self-rated health						
Good	101(24.6)	77(35.2)	14(14.3)	10(10.6)	40.276	<0.001^***^
Fair	115(28.0)	61(27.8)	35(35.7)	19(20.2)		
Poor	195(47.4)	81(37.0)	49(50.0)	65(69.2)		
Barthel index	411(100)	70(65,85)	70(55,80)	60(50,75)	33.568^#^	<0.001^***^

^#^*H* value; *P < 0.05, **p < 0.01, ***p < 0.001.

### 3.3 Latent profiles analysis

Latent profile models were fitted using the five dimensions of demoralization. The model fit indices are presented in Table 2. As the number of profiles increased, the values of AIC, BIC, and aBIC gradually decreased. The entropy value was highest in the 2-profile model. The BLRT was statistically significant (*p* < 0.05) for all models; however, the LMR was non-significant (*p* > 0.05) for the 5-profile and 6-profile models, indicating their unsuitability.

**Table 2:** Fit statistics for each profile structure.

Profile	AIC	BIC	aBIC	Entropy	LMR (*P*)	BLRT (*P*)	Class probability
1	5546.188	5586.374	5554.642	—	—	—	—
2	4002.322	4066.619	4015.848	0.955	<0.001	<0.001	70.8/29.2
3	3606.122	3694.531	3624.720	0.910	0.0009	<0.001	53.3/23.8/22.9
4	3461.071	3573.591	3484.742	0.873	0.0146	<0.001	28.2/33.3/18.0/20.5
5	3399.357	3535.989	3428.100	0.866	0.0510	<0.001	30.9/16.3/26.0/10.7/16.1
6	3366.613	3527.357	3400.429	0.866	0.1452	<0.001	10.2/25.8/31.6/6.8 /10.0/15.6

AIC, Akaike Information Criterion; BIC, Bayesian Information Criterion; aBIC, the sample-size-adjusted BIC; LMR, Lo–Mendell–Rubin corrected Likelihood Ratio Test; BLRT, Bootstrap-based Likelihood Ratio Test.

When comparing the 3-profile and 4-profile models, it was found that the entropy and LMR value of the 4-profile model were worse, while the decreases in AIC, BIC, and aBIC values were limited. Considering the practical relevance and interpretability of the classification results, the 3-profile model was ultimately selected as the best-fitting solution. For the 3-profile model, the entropy value was 0.910, and the proportion of individuals in each profile was appropriate. Furthermore, as shown in Table 3, the average latent class probabilities ranged from 0.936 to 0.968, indicating high classification accuracy and further supporting the appropriateness of the 3-profile model.

**Table 3:** Attribution probabilities for each latent profile of subjects.

Class	Profile 1	Profile 2	Profile 3
1	0.964	0.036	0.000
2	0.052	0.936	0.012
3	0.000	0.032	0.968

**Figure 1:**
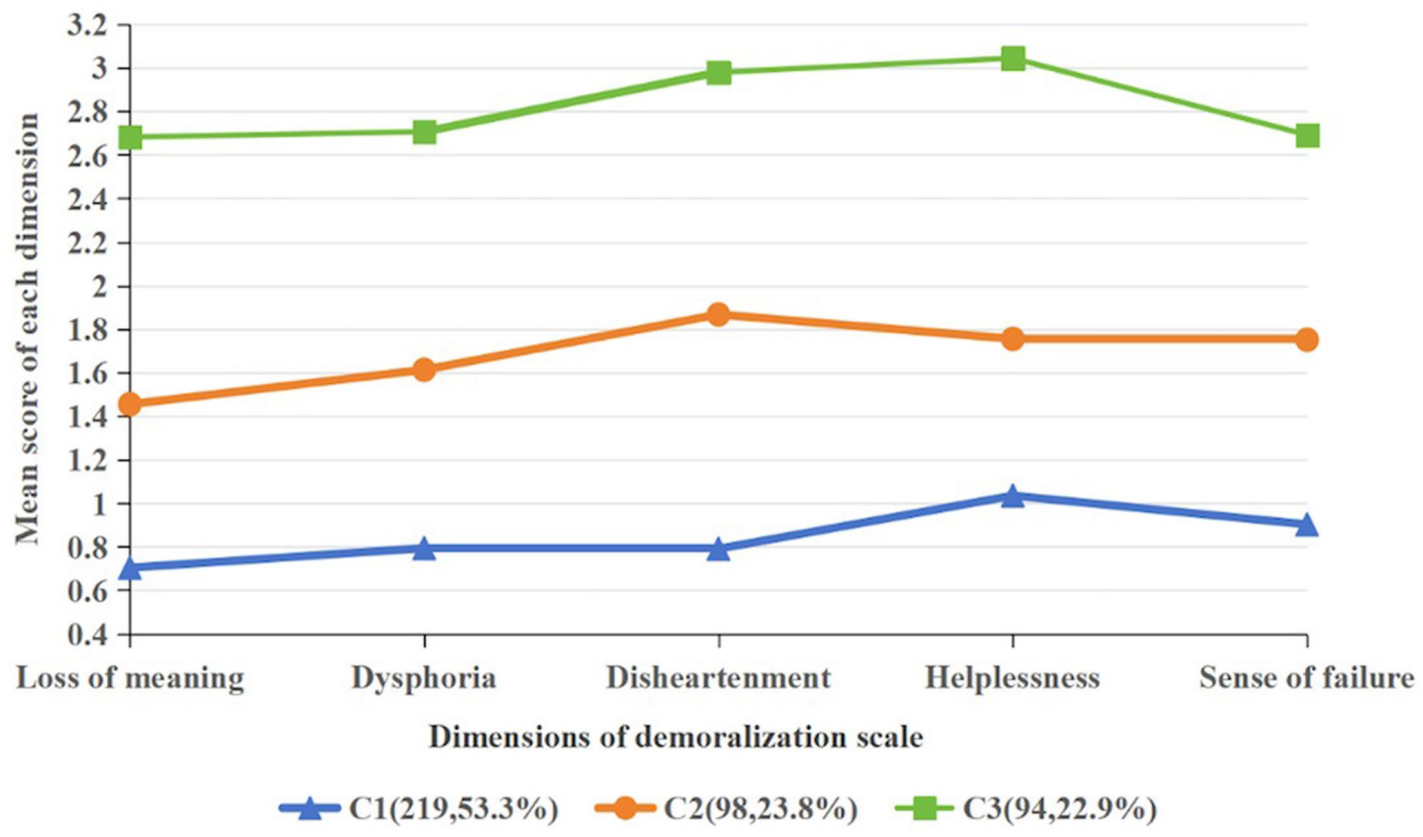
Three subtypes of demoralization syndrome based on the latent profile analysis results.

Figure 1 depicts the mean profile plot of the three latent classes across the five dimensions of demoralization. These classes were named according to their distinctive characteristics: Class 1 (C1), comprising 53.3% of the sample, demonstrated significantly lower scores across all dimensions and was labeled the Well-Adapted Group. Class 2 (C2), accounting for 23.8%, exhibited the highest mean scores on the dimensions of disheartenment and helplessness, and was designated the Disheartened-Helpless Group. Class 3 (C3), representing 22.9%, showed significantly higher scores across all dimensions and was named the Fully Demoralized Group.

### 3.4 Univariate analysis

As shown in Table 1, statistically significant differences were observed across the different latent demoralization profiles among older adults with disabilities regarding religious affiliation, educational level, place of residence, living arrangement, pension status, monthly household income per capita, primary caregiver, healthcare payment method, disability duration, cause of disability, Barthel Index score, and self-rated health status (*p* < 0.05).

### 3.5 Multinomial logistic regression analysis

We categorized demoralization syndrome into three latent profiles, designating the Well-Adapted Group as the reference category. Variables that showed statistical significance in the univariate analysis were then included in a multinomial logistic regression model to identify factors associated with membership in each latent demoralization profile. Multinomial logistic regression automatically handles categorical data without requiring additional dummy variable coding. The value assignments for each variable were as follows: (1) Religion: 1 = No; 2 = Yes; (2) Educational level: 1 = Primary school or below; 2 = Junior school; 3 = High school or vocational school; 4 = College/University or higher; (3) Residence: 1 = Rural; 2 = Towns; 3 = Cities; (4) Living status: 1 = Living alone; 2 = With families; 3 = Other; (5) Pension status: 1 = No; 2 = Yes; (6) Monthly household income per capita (RMB): 1 = ≤3,000; 2 = 3,001–5,000; 3 = ≥5,001; (7) Primary caregiver: 1 = Self-care; 2 = Spouse/Children; 3 = Other; (8) Healthcare payment method: 1 = Self-pay; 2 = Resident insurance; 3 = Employee insurance; (9) Disability duration: 1 = 6 months~; 2 = 1 year~; 3 = 3 years~; 4 = 6 years~; 5 = ≥9 years; (10) Cause of disability: 1 = Congenital disability; 2 = Illness; 3 = Accident; 4 = Aging; (11) Self-rated health: 1 = Good; 2 = Fair; 3 = Poor. The Barthel Index scores were entered as their original values.

The results of the multinominal analysis are presented in Table 4. Compared to the Well-Adapted Group, older adults with lower educational levels, lower monthly household income per capita, and whose primary caregivers were their spouses or children were more likely to belong to the Disheartened-Helpless Group. Those with lower monthly household income per capita, out-of-pocket healthcare payments, a disease-related cause of disability, and who were their own primary caregivers were more likely to belong to the Fully Demoralized Group. Conversely, older adults who lived alone, had no religious affiliation, had a longer duration of disability, better self-rated health, and had less severe disability were more likely to belong to the Well-Adapted Group.

**Table 4:** Results of the multinomial logistic regression analysis of latent demoralization profiles among older adults with disabilities (*n* = 411).

Variables		C2				C3			
		OR	95%CI		*P*	OR	95%CI		*P*
			LLCI	ULCI			LLCI	ULCI	
Religion	No	0.462	0.220	0.972	0.042	2.104	0.774	5.721	0.145
Education level	Primary school or below	3.812	1.218	11.933	0.022	2.751	0.878	8.621	0.082
	Junior school	2.301	0.831	6.377	0.109	0.841	0.307	2.304	0.736
	High school or Vocational school	2.752	1.019	7.431	0.046	2.142	0.879	5.219	0.094
Living	Rural	1.447	0.598	3.498	0.413	1.828	0.667	5.010	0.241
	Towns	0.884	0.390	2.006	0.769	1.965	0.843	4.578	0.117
Living status	Living alone	1.308	0.128	13.377	0.821	0.177	0.037	0.837	0.029
	With families	1.133	0.119	10.747	0.914	0.278	0.071	1.085	0.065
Pension status	No	1.223	0.636	2.351	0.547	1.068	0.527	2.167	0.855
Personal monthly income (RMB)	≤3,000	4.085	1.770	9.432	0.001	2.622	1.084	6.344	0.032
	3,001–5,000	1.948	0.897	4.233	0.092	1.479	0.694	3.153	0.311
Primary Caregiver	Self-care	2.194	0.663	7.258	0.198	3.937	1.169	13.259	0.027
	Spouse/ Children	3.042	1.149	8.056	0.025	2.026	0.837	4.909	0.118
Health insurance type	Self-pay	1.628	0.511	5.186	0.410	3.801	1.166	12.386	0.027
	Resident insurance	1.006	0.424	2.385	0.990	1.211	0.487	3.007	0.681
Disability duration	6 months~	1.610	0.471	5.507	0.447	0.712	0.193	2.623	0.610
	1 year~	0.860	0.274	2.705	0.797	1.097	0.388	3.103	0.861
	3 years~	0.899	0.342	2.365	0.829	0.447	0.173	1.154	0.096
	6 years~	0.988	0.359	2.722	0.981	0.332	0.124	0.891	0.029
Cause of disability	Congenital disability	1.095	0.071	16.997	0.948	3.873	0.379	39.588	0.254
	Illness	1.926	0.964	3.849	0.063	2.856	1.290	6.324	0.010
	Accident	0.941	0.438	2.021	0.875	1.159	0.473	2.837	0.747
Self-rated health	Good	0.498	0.208	1.193	0.118	0.360	0.138	0.936	0.036
	Fair	1.169	0.595	2.294	0.650	0.673	0.320	1.415	0.296
Barthel index		0.977	0.953	1.001	0.064	0.944	0.919	0.969	<0.001

C2, Disheartened-Helpless Group; C3, Fully Demoralized Group.

### 3.6 Comparison of active aging scores across demoralization profiles

The Kruskal–Wallis *H* test was employed to compare differences in active aging scores among the distinct demoralization profiles, with the results presented in Table 5. Statistically significant differences were found in the total active aging scores across the profiles (*H* = 275.349, *p* < 0.001). The Fully Demoralized Group had the lowest mean rank, followed by the Disheartened-Helpless Group, while the Well-Adapted Group demonstrated the highest mean rank, indicating the most favorable level of active aging.

**Table 5:** Comparison of active aging among different demoralization subgroups.

Profile	Active aging [*M* (P25, P75)]	Mean rank
Well-Adapted Group	95(88,105)	295.04
Disheartened-Helpless Group	75(70,79)	133.35
Fully Demoralized Group	68(64,73)	74.31
*P*	<0.001	
H	275.349	

## 4 Discussion

### 4.1 Prevalence of demoralization subtypes

The results revealed a demoralization syndrome prevalence of 49.1% among older adults with disabilities, with a median total score of 29 (20, 51). This indicates that nearly half of this population experiences varying degrees of demoralization. This phenomenon may be attributed to the unique challenges associated with disability. The dependence in physical functioning, constriction of social networks, and regression of social roles that often accompany disability can easily trigger negative emotions (39), feelings of helplessness, and doubts about self-worth (40). These findings underscore the urgent need to integrate psychosocial support into the core care plan for disabled older adults, alongside basic life care. Early assessment, emotional support, and empowerment interventions are essential to enhance their sense of control over life, thereby alleviating demoralization and improving overall well-being.

Using Latent Profile Analysis, this study identified three distinct profiles: the Well-Adapted Group, the Disheartened-Helpless Group, and the Fully Demoralized Group. The Well-Adapted Group, characterized by low scores across all demoralization dimensions, suggests the presence of protective factors against psychological distress. Investigating these factors can guide families and healthcare providers in strengthening resources to help older adults cope with disability. The Disheartened-Helpless Group likely represents an early or middle stage of demoralization. These individuals may desire participation but face functional limitations, and wish for social connection but have shrinking networks, exhibiting prominent features of disheartenment and helplessness. This highlights the necessity for healthcare professionals to routinely screen for demoralization to enable early identification and intervention. In contrast, the Fully Demoralized Group experiences profound hopelessness and helplessness, potentially the result of long-term chronic progression, necessitating more intensive and systematic medical and psychological interventions. Notably, scores for helplessness were relatively high across all three profiles, reflecting a pervasive need for multifaceted support among disabled older adults. Therefore, future research employing qualitative interviews is warranted to gain deeper insights into their needs and develop more targeted support services.

### 4.2 Factors associated with demoralization profiles

#### 4.2.1 Fully demoralized group

The results of the multinomial logistic regression analysis indicated that older adults with disabilities who had a monthly household income per capita below 3,000 RMB and out-of-pocket healthcare payments were at a higher risk of belonging to the Fully Demoralized Group. Economic status directly impacts the resources and strategies available to older adults and their families for coping with disability (41). Research has shown that low-income groups often face barriers in accessing healthcare services, social activities, and psychological support (42). Furthermore, out-of-pocket healthcare payments can create a resource crowding-out effect, reducing both the willingness and ability to invest in mental health services (43). This finding suggests that establishing robust economic security is a structural prerequisite for mitigating such psychosocial risks. Notably, China has already initiated pilot programs providing consumption subsidies for care services to older adults with disabilities (44), laying a practical foundation for developing a nationwide specialized subsidy system. To further enhance the level of protection, subsequent policies could focus on expanding the coverage of existing subsidies to include essential items for disabled older adults, such as rehabilitation assistive devices and professional psychological counseling services. Additionally, it is recommended to establish a dynamic adjustment mechanism for subsidy standards based on the price index and the market costs of long-term care services, thereby more comprehensively alleviating the psychological distress caused by economic pressure. Furthermore, the practice in the Hong Kong Special Administrative Region, where the Comprehensive Social Security Assistance (CSSA) scheme provides additional cash subsidies to older adults choosing residential care in Guangdong (45), serves as an important reference for the mainland in exploring flexible use of subsidy funds.

The findings revealed that older adults whose disability was caused by disease were more likely to belong to the Fully Demoralized Group. The progression of disease is often insidious and unpredictable. Whether the onset is acute or chronic, it frequently leads to a high degree of disability. Older adults often lack psychological preparedness for the outcome of functional decline, making them prone to emotional volatility and maladaptive responses (17). Concurrently, the physical symptoms associated with illness and the accompanying socioeconomic burden deplete an individual’s coping resources, thereby increasing susceptibility to feelings of hopelessness and meaninglessness (46). Therefore, it is imperative to vigorously promote an integrated hospital-community-family care system. By clarifying the responsibilities of institutions at all levels in rehabilitation referrals and care planning, the continuity and coordination of services can be ensured. To this end, policy efforts should shift toward establishing a mechanism for proactively identifying high-risk individuals, incorporating “demoralization screening” into routine services, and supporting it with simplified tools, inter-facility connectivity and referral pathways, along with corresponding incentive policies. This will effectively embed early identification of psychological issues into routine primary-level practices.

It is noteworthy that older adults who primarily manage their daily care independently also showed a higher risk of belonging to the Fully Demoralized Group. According to self-efficacy theory (47), an individual’s belief in their own capabilities is paramount. For such individuals, despite retaining some self-care capacity, the subjective experience of functional impairment intensifies over time. This implies continual exposure to negative somatic perceptions in daily life, which gradually erodes their sense of self-efficacy (48). In the absence of necessary caring support and guidance for fostering a positive self-perception, these older adults are more susceptible to developing negative self-assessments, reinforcing feelings of discouragement and failure, and ultimately descending into demoralization (49). Therefore, public policies must transition from a “disability-level-centered” to a “psychosocial-risk-centered” assessment and resource allocation framework, explicitly including older adults with mild to moderate disabilities yet fragile support systems as a key target population. It is recommended to promote the establishment of integrated community centers that consolidate daycare, rehabilitation, and social activities. These centers would provide services and social support for older adults with varying levels of disability, thereby combating social isolation and rebuilding their sense of self-efficacy.

#### 4.2.2 Disheartened-helpless group

The results indicate that lower educational levels and a household monthly income per capita below 3,000 RMB (OR = 4.085) increase the likelihood of belonging to the Disheartened-Helpless Group. Lower education levels and poor economic status may constrain an individual’s ability to cope with disability, reflecting insufficient self-help capabilities (50). Simultaneously, these factors can impede access to external assistance and limit the comprehension of available support information, representing restricted access to help from others (51). Being trapped in the negative emotions associated with disability while unable to obtain adequate support exacerbates feelings of discouragement and helplessness among older adults. Therefore, in addition to providing corresponding financial subsidies, healthcare providers and community workers should prioritize the implementation of “community health literacy” education programs tailored for older adults with lower educational levels. These programs should deliver knowledge on emotion management and help-seeking skills in an accessible manner. Concurrently, simplified and practical social service information guides should be provided to proactively establish effective channels for external support, thereby improving their state of demoralization.

Moreover, “household monthly income per capita below 3,000 RMB” also serves as a significant risk factor for the Fully Demoralized Group (OR = 2.622), revealing that financial pressure constitutes a pervasive and fundamental threat to the psychological well-being of older adults with disabilities. The key distinction lies in the differing synergistic factors: within the Disheartened-Helpless Group, the combination of financial pressure and low educational levels primarily indicates a deficiency in psychosocial resources, such as coping capacities and help-seeking channels. In contrast, for the Fully Demoralized Group, its synergy with factors like out-of-pocket healthcare payments more directly reflects a lack of structural support. Therefore, while universal economic security policies are necessary, precise mental health interventions must further identify these distinct risk combination patterns.

The findings also revealed that having a child or spouse as the primary caregiver is a significant factor influencing demoralization in older adults with disabilities. This may be because reliance on care from others often indicates poorer functional capacity and/or the presence of social support, but it can also signify a diminished sense of personal control over one’s own life (52, 53). Over time, this dynamic can foster feelings of discouragement regarding life. Furthermore, long-term care receipt can intensify feelings of guilt in older adults, leading to avoidant behaviors that indirectly suppress the expression of emotional needs (54, 55). The presence of social support, coupled with a reluctance to express these needs, may paradoxically exacerbate feelings of helplessness. Therefore, it is recommended that relevant policies be extended to consider the caregiver’s perspective by accelerating the development of “respite services” across different regions, thereby alleviating feelings of guilt among older adults. Additionally, promoting “caregiver communication training” through community and medical institutions would encourage caregivers to appropriately empower older adults with disabilities, enhance daily emotional communication, and foster a healthier, more supportive family care environment.

#### 4.2.3 Well-adapted group

The results indicated that having no religious affiliation was associated with a higher likelihood of belonging to the Well-Adapted Group. This finding differs from the common perception in some studies that views religious belief as a protective factor (56). In the context of Chinese culture, those without religious beliefs may find that strong family and community support serves as a substitute source of meaning and backing, thereby facilitating their psychological adaptation (57). However, in our study, religious belief was associated with poorer psychological adaptation. A possible explanation is that the psychological distress associated with disability (such as demoralization) may drive older adults to seek religious faith as both a coping mechanism and a source of solace, reflecting how poor psychological conditions can motivate religious behavior (58).

The results demonstrated that less severe disability and better self-rated health were associated with a higher likelihood of belonging to the Well-Adapted Group. Milder disability implies better physical functioning and fewer life restrictions, providing an objective foundation for maintaining participation and a positive mindset (59). Furthermore, favorable self-rated health not only reflects one’s objective physical condition but also indicates a strong sense of health-related control and positive cognitive appraisal (60), thereby enabling more effective coping with the challenges posed by disability. Additionally, within the context of China’s filial piety culture, the ability to live alone is often perceived as an objective indicator of relatively mild disability. This perception indirectly reinforces the older adult’s sense of independence and self-worth, collectively contributing to the maintenance of a positive psychological state.

The results indicated that a disability duration of 6–9 years was associated with a higher likelihood of belonging to the Well-Adapted Group. According to crisis adaptation theory (61), this duration suggests that the individual has navigated through the acute, distressing phase following the onset of disability and has entered a relatively stable long-term adaptation stage. This period provides sufficient time to adjust life rhythms, reconstruct self-identity, and develop personalized and effective coping strategies.

Analysis of these influencing factors clearly highlights the significant roles that disability severity and individual health status play in the development of demoralization. It is recommended that healthcare professionals provide specialized rehabilitation services to maintain or improve functional abilities, while also prioritizing early psychological assessment and support. Family members should avoid adopting an overly “paternalistic caregiving” approach. Instead, they should encourage the exercise of remaining functions and emphasize emotional communication and psychological comfort to mitigate demoralization levels in older adults.

#### 4.2.4 Association between demoralization and active aging

This study reveals a significant association between demoralization profiles and active aging levels among older adults with disabilities, confirming that psychological state is a key predictor of behavioral and developmental outcomes in later life. From an internal mechanism perspective, demoralization directly erodes the psychological foundations of the three pillars of active aging. A sense of meaninglessness and lack of motivation diminish the willingness for social participation, leading to social withdrawal and a loss of a sense of value (18); pessimistic expectations regarding health outcomes reduce treatment adherence and self-management willingness, accelerating functional decline (22, 62). While active aging emphasizes providing security for older adults, the subjective sense of incompetence and failure associated with demoralization hinders the initiative to seek out and effectively utilize available social support resources, thereby exacerbating life uncertainties (63). At its core, active aging requires individuals to exert agency, and demoralization constitutes a negative force that undermines this very agency.

In contrast, even in the presence of functional limitations, the low-demoralization group maintained relatively high levels of active aging. This finding provides a critical entry point for practical interventions. It is recommended to leverage professional expertise to deliver stratified and targeted support: structured psychoeducational interventions should be prioritized for the high-demoralization group to rebuild a sense of meaning and control. Concurrently, graded social participation activities should be designed, progressing from companion-based socialization to more constructive community involvement, to systematically enhance active aging levels.

### 4.3 Limitations

This study has several limitations. First, the use of convenience sampling from a single hospital limits the sample’s representativeness, which may constrain the generalizability of the findings. Future research could employ multi-stage stratified random sampling and increase the sample size to enhance representativeness. Second, the cross-sectional design precludes the determination of longitudinal trajectories of demoralization and the inference of causal relationships. Future studies should investigate the dynamic trends of demoralization in this population to improve understanding of their psychological health over time. Third, the primary variables were measured mainly using self-reported questionnaires, which are susceptible to potential recall bias. Future research would benefit from incorporating a combination of self-report and observer-rated tools, as well as both subjective and objective indicators, to strengthen the robustness of the results.

## 5 Conclusion

This cross-sectional study revealed that nearly half of the older adults with disabilities experienced demoralization, and their demoralization characteristics exhibited significant population heterogeneity, which can be categorized into three distinct profiles: the Well-Adapted Group, the Disheartened-Helpless Group, and the Fully Demoralized Group. Significant differences were observed in demographic characteristics and active aging levels across these profiles, with the Fully Demoralized Group requiring the most urgent attention. The patterns of association between variables identified in these analyses suggest that the assessment and management of psychological distress in this population should adopt a profile-specific evaluation and precision intervention model. Healthcare providers should prioritize the mental health of older adults with disabilities, conduct regular demoralization screenings, and provide differentiated support based on their specific profile membership. Future research should focus on developing and validating interventions tailored to this classification system, aiming to fundamentally enhance the physical and mental well-being and quality of life of older adults with disabilities.

## Data Availability

The raw data supporting the conclusions of this article will be made available by the authors, without undue reservation.
